# Multivariate time-series forecasting of liver biomarkers from longitudinal lifestyle data for nonalcoholic steatohepatitis detection

**DOI:** 10.1093/jamiaopen/ooag046

**Published:** 2026-04-11

**Authors:** Sumaiya Afroz Mila, Sandip Ray

**Affiliations:** Department of Electrical and Computer Engineering, University of Florida, Gainesville, FL 32611, United States; Department of Electrical and Computer Engineering, University of Florida, Gainesville, FL 32611, United States

**Keywords:** deep learning, machine learning, time-series modeling, nonalcoholic fatty liver disease (NAFLD), nonalcoholic steatohepatitis (NASH)

## Abstract

**Objectives:**

To develop a machine learning method that estimates future liver biomarkers’ values from longitudinal lifestyle (diet, activity) data for early detection of nonalcoholic steatohepatitis (NASH).

**Materials and Methods:**

The method in this study is developed utilizing the nonalcoholic fatty liver disease adult dataset, by National Institute of Diabetes and Digestive and Kidney Diseases, a real-world dataset representative of common electronic health records in the United States. We have developed time-series Machine Learning/Deep Learning and tree-based models to forecast future values for liver biomarkers, identified the minimum requirement of initial data points for optimal forecasting performance, and developed time-series classifier models for detecting NASH from longitudinal lifestyle data and initial biomarker values.

**Results:**

Our experiments show that lifestyle-informed forecasting models, such as Attention-long short-term memory and TimeSeriesForestRegressor accurately predict future biomarker trajectories with as few as 2 observed timepoints (prediction error as low as 0.62), and NASH classifiers trained on these *Fo*recasting liver *Bi*omarkers (*FoBi*) estimated biomarkers achieve performance (accuracy 86%) comparable to or exceeding existing biopsy-aligned methods.

**Discussion:**

The proposed approach, *FoBi*, is the first method to forecast liver biomarker trajectories from lifestyle data and demonstrate that both observed and model-estimated biomarkers can support effective NASH detection in real-world clinical settings.

**Conclusion:**

Lifestyle-driven biomarker forecasting offers a promising, minimally invasive foundation for early NASH detection and long-term disease management, reducing dependence on frequent laboratory testing and biopsy-aligned measurements.

## Introduction

Nonalcoholic fatty liver disease (NAFLD) affects nearly one-third of adults in the world, where 10% affected individuals in the United States are lean and 69% of those individuals are unaware of their condition.^1–3^ Nonalcoholic fatty liver disease represents a spectrum of progressive liver disease, beginning with simple steatosis, which can advance to nonalcoholic steatohepatitis (NASH), fibrosis, cirrhosis, and, ultimately, hepatocellular carcinoma.^4^ Among these stages, NASH is the most clinically critical transition, since it is the last reversible and curable stage of liver disease.^5^ However, undetected and untreated NASH can accelerate fibrosis progression since it reflects sustained hepatocellular injury and inflammation.^6^ Detecting NASH early is essential because timely lifestyle or therapeutic interventions can meaningfully alter disease trajectory; however, NASH remains largely underdiagnosed, and most patients progress silently until irreversible damage occurs.

Current clinical methods for NASH detection are limited. Liver biopsy is the diagnostic gold standard but is invasive, expensive, mostly reserved for advance stage patients.^7^ Semi-invasive or noninvasive approaches like serum biomarkers, imaging modalities (ultrasound, elastography), and composite scoring systems (eg, FIB-4, NAFLD Fibrosis Score) are not part of routine clinical checkups.^8^^,^^9^ More recent advances, such as machine learning models trained on biopsy-aligned laboratory features (eg, the NASHmap algorithm^10^^,^^11^) achieve promising performance but depend on precisely collected blood marker panels at the biopsy visit and do not utilize the longitudinal lifestyle behavior. Thus, current solutions either require invasive procedures or rely heavily on laboratory measurements that are rarely collected routinely in real life.

Lifestyle information, such as diet, physical activity, weight patterns are continuously observable, highly predictive of metabolic health, and feasible to monitor outside the clinic. Prior studies have demonstrated associations between lifestyle factors and liver health outcomes; however, many existing approaches primarily perform population-level association or trajectory analyses with single-timepoint liver assessments (eg, de Brito et al.^12^) or develop static predictive classifiers using biopsy-aligned laboratory panels,^10^^,^^11^ or implement partial physiology-informed modeling frameworks.^13^ These approaches do not explicitly forecast individualized longitudinal biomarker trajectories under sparse observation settings from lifestyle information. *FoBi* differs by modeling liver monitoring as a longitudinal forecasting problem: we predict future biomarker trajectories from lifestyle time series with limited initial lab values and then quantify how these estimated trajectories support downstream NASH detection. The role of structured lifestyle (diet and activity) time-series data in forecasting future liver biomarker dynamics remains relatively underexplored. If lifestyle information can be transformed into reliable predictions of future biomarker trajectories, clinicians could monitor progression remotely, reduce reliance on frequent laboratory assessments and invasive procedures, and detect disease progression earlier using data that patients can self-report or track automatically.

To address this gap, we propose *FoBi* (*Fo*recasting liver *Bi*omarkers from longitudinal lifestyle data), a longitudinal multivariate time-series forecasting framework designed to evaluate whether structured lifestyle information, combined with a limited number of initially observed biomarker values, can be used to (1) forecast future liver biomarker trajectories and (2) detect NASH, thereby supporting progression monitoring following NAFLD diagnosis.

Unlike prior work that either (1) performs association analysis between lifestyle (focused on physical activity) trajectories and single-timepoint liver outcomes^12^ or (2) develops static predictive classifiers based on biopsy-aligned laboratory panels,^10^^,^^11^  *FoBi* introduces a unified 2-phase framework that explicitly models the temporal evolution of liver biomarkers as an intermediate forecasting task before downstream disease classification. A structured comparison between representative prior approaches and *FoBi* is summarized in Table 1. This structured 2-step decomposition between biomarker trajectory estimation and NASH detection enables evaluation of how much prior clinical supervision is minimally required to maintain reliable disease detection. This work makes the following 4 key contributions:

**Table 1:** Detailed methodological comparison between prior NASH detection approaches and *FoBi*.

Method	Longitudinal	Lifestyle	Forecasts	Two-stage	Varying clinical	Biopsy
	data	input^a^	biomarkers	design	supervision	alignment
**Trajectory/Association studies (eg, de Brito et al.^12^)**	√	√	×	×	×	×
**Static biopsy-aligned ML models (eg, NASHmap^10^^,^^11^)**	×	×	×	×	×	√
**Partial physiology-informed models (eg, TWIN-SCAN^13^)**	√	*Partial*	*Partial*	×	×	×
***FoBi* (proposed)**	√	√	√	√	√	×

a Lifestyle input indicates whether structured diet/physical activity/weight time series are used as model inputs. Biopsy alignment indicates whether biopsy-day laboratory alignment is required by the method.

- Introducing a 2-phase framework that decomposes NASH detection into longitudinal multivariate biomarker forecasting followed by disease classification, enabling explicit evaluation of temporal biomarker dynamics.
- Systematically analyzing sparse supervision by varying the number of initially observed biomarker timepoints to determine the minimal clinical information required for stable performance.
- Designing a hybrid evaluation setting comparing ground-truth vs *FoBi*-estimated biomarker trajectories to quantify how forecasting uncertainty propagates into downstream NASH classification.
- Demonstrating NASH detection feasibility from *FoBi*-estimated biomarker trajectories in both real-world nonbiopsy-aligned longitudinal settings and biopsy-aligned configurations.

## Materials and methods

**Figure 1:**
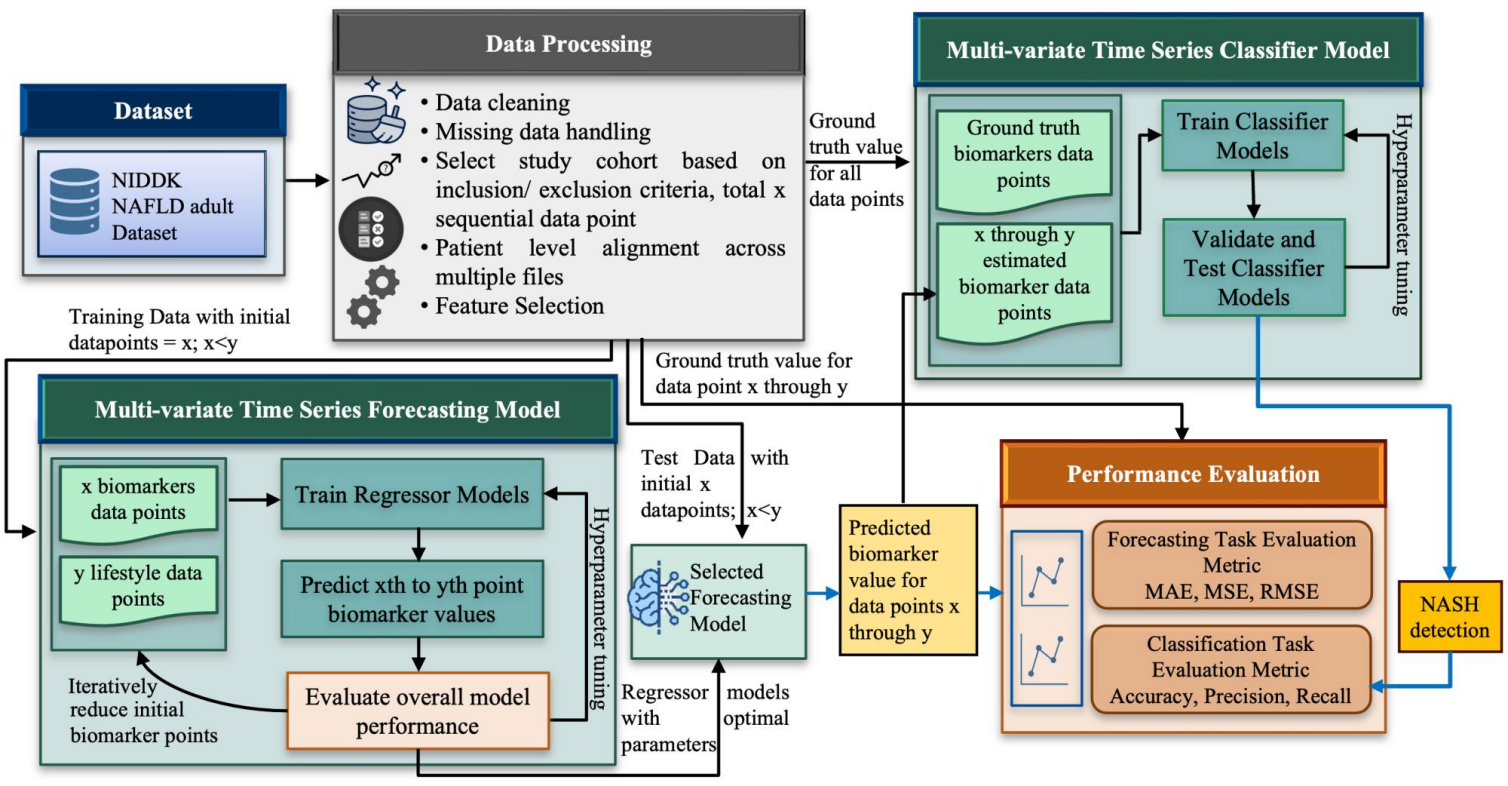
Workflow for forecasting biomarkers and NASH detection.

Building upon the 2-phase design described in the “Introduction” section, our *FoBi* approach for early NASH detection is designed to leverage lifestyle information and minimal prior biomarker information. This work uses the NAFLD dataset^14^ by National Institute of Diabetes and Digestive and Kidney Diseases (NIDDK) from which we extract “continuously trackable” lifestyle information, develop the *FoBi* method that estimates biomarker values for early NASH detection. Figure 1 demonstrates the high-level overview of the methodology for developing *FoBi*. This comprehensive methodology allows us to not only fully utilize lifestyle data but also develop a stable noninvasive statistical NASH detection method only from lifestyle data.

1. Data preprocessing: Common critical issues found in real-world datasets include missing values, unstructured data, and mislabeled or unlabeled data. These issues must be carefully considered and resolved during the data preprocessing step. We ensure that the raw dataset is properly prepared and processed to make it compatible for the subsequent analysis and experiments by addressing and resolving these key issues.
2. Machine learning model overview: To evaluate the feasibility of noninvasive NASH detection primarily from lifestyle data and a few initial observation of biomarker data, we developed 2 complementary machine learning frameworks under the *FoBi* methodology. The first framework focuses on multivariate time-series forecasting, where models are trained to predict future biomarker values based on patients’ lifestyle attributes and a limited number of initial biomarker observations. This approach enables the estimation of longitudinal biomarker trajectories using noninvasive, continuously trackable data. We vary the number of initially observed biomarker data points to identify the minimum number of biomarker information required for optimal future estimation. The second framework addresses multivariate classification, in which models are trained to distinguish NASH from non-NASH conditions using both, ground-truth and *FoBi*-estimated biomarker values along with lifestyle sequences. Together, these 2 frameworks form a unified pipeline that first forecasts the likely progression of key liver biomarkers and then applies those estimated trends for early stage disease classification.

The methodological novelty of *FoBi* lies in its 2-stage decomposition of the NASH detection problem. Instead of directly mapping lifestyle features to NASH labels, we first model liver biomarker dynamics as a multivariate time-series forecasting task and then evaluate whether estimated biomarker trajectories preserve sufficient discriminative signal for disease classification. This intermediate forecasting layer enables (1) assessment of minimal biomarker supervision requirements, (2) explicit evaluation of temporal generalization, and (3) controlled comparison between ground-truth and estimated biomarker-driven NASH detection. This design differs from prior approaches that rely on direct feature-to-label mappings without explicitly modeling intermediate biomarker dynamics.

### Data preprocessing

For this work, we use the NAFLD adult dataset (version 4) by NIDDK which consist of 1410 adult participants with diagnosed NAFLD observed over a 4-year period (2004-2009).^14^ The patients are classified based on their liver status as NASH or non-NASH as confirmed through liver biopsy and histological assessment. Available data include demographic, histological, clinical biomarkers, diet, physical activity information of the enrolled participants where data are collected for up to 4 years of enrollment. Patients with other liver diseases (eg, viral hepatitis, alcoholic cirrhosis) or excessive alcohol consumption were excluded. Data collection interval is as follows: during screening (first 2 visits), 24 weeks after enrollment (annotated as f024), 48 weeks after enrollment (f048), and at yearly intervals thereafter (a maximum of 7 visits where the latter visits are referred to as f096, f144, f192). (Ethical considerations, IRB approval, and data privacy: Use of the NIDDK NAFLD Adult Database for this study was approved by the University of Florida Institutional Review Board (Protocol IRB202500643) ensuring compliance with all ethical standards for handling limited-access human subject data. The dataset, obtained from the NIDDK Central Repository, may include information that could potentially identify participants; however, no reidentification or patient-level linkage was performed. All analyses were conducted in accordance with the UF Health Information Technology Services data security and privacy policies.)

Missing or incomplete values are common challenges in real-world datasets, requiring certain processing steps to prepare the data for a specific task. The irregularities or flaws in real-world raw data can adversely impact the outcomes of a machine learning model, leading to suboptimal model performance. Hence, we perform data processing techniques as described in the following subsections to prepare the NIDDK dataset for our 2 phase experiment design.

#### Appropriate data files and features selection

The NIDDK dataset has an extensive information stored for each participant. However, since the goal of our work is to analyze the feasibility of NASH detection once NAFLD has been diagnosed, only from the patient’s longitudinal lifestyle data, we only focus on the files containing diet, physical activity, laboratory test result, central histology, and registration information of the patients. Each of these files mentioned above has multiple features. Table 3 shows the final set of features selected across various files. From now on we will use the acronyms alanine transaminase (ALT), aspartate transaminase (AST), alkaline phosphatase (ALP), and gamma glutamyl transferase (GGT) to refer to the liver biomarkers. Note that, id and visit information are used to properly align patient’s information for a specific visit across different files. We keep our feature space minimal with absolutely basic dietary and activity information, basic biomarkers^15^ that are used to detect NAFLD/NASH in the early stage and can be collected through minimally invasive procedure.

**Table 2:** Patient distribution across different data files.

File	Total unique patients^a^	**Percentage of patients with**			
		2 samples	3 samples	4 samples	5 samples
**Diet information (DI)**	1085	28.76%	23.78%	14.01%	14.29%
**Physical activity (PA)**	1191	27.54%	22.59%	12.93%	14.78%
**Laboratory tests (LR)**	1410	26.17%	21.99%	15.04%	15.74%
**Central histology (CR)**	1066	This file is only used for extracting target status			
**Registration (RG)**	1277	This file is only used for age, gender, ethnicity distribution			

a Common unique patients across 5 files = 938; common unique patients with minimum 2 visits across 5 files = 825.

**Table 3:** Summary of missing/null values across features.

Feature	Missing_Count	Missing_Percentage
**id, visit, age, gender**	0	0
**Weight**	276	9.47
**Height**	280	9.61
**Activity_level**	183	6.28
**Hours/day in this activity_level**	183	6.28
**Hours/day spent sitting**	183	6.28
**Daily_calories**	269	9.23
**Protein**	269	9.23
**Carb**	269	9.23
**Fat**	269	9.23
**Total_bilirubin**	27	0.93
**Direct_bilirubin**	125	4.29
**Albumin**	40	1.37
**AST**	27	0.93
**ALT**	25	0.86
**ALP**	33	1.13
**GGT**	173	5.94

#### Cohort selection and patient alignment across multiple data files

Patients from the NIDDK database with confirmed NASH or non-NASH status by biopsy (extracted from central histology [CR]) were included in this study. The NIDDK dataset has an initial of 1410 patients. First, we identify the common patients (938 participants) across the first 3 files of our interest mentioned in Table 2. Since our goal is to analyze forecasting performance and NASH detection from longitudinal data, we keep patients who have atleast 2 samples/follow-up information (825 participants have data collected from minimum 2 visits), and discard the patients who only have one data entry. Unique patient id and visit code were used to align patients across the 5 datasets. Table 2 shows the summary of unique patients in different data files, common patients across these files and the percentage of patients with different amount of follow-up information. Central histology and registration files are only used to identify the target state of NASH/non-NASH, ethnicity, age, and gender distribution of the participants.

#### Handling missing data

We extracted dietary, activity, and test result information across different data files, aligned information of the selected feature for the patients across their different follow-up visits, and stored the processed dataset. This processed dataset still has some real-world limitations, such as missing values/null values. Table 3 shows the summary of total count of missing/null values for each feature and the percentage of it. We process each column/feature in the dataset for resolving missing/null data assuming the following scenarios:

1. value missing at the screening day,
2. value missing at the last follow-up visit,
3. random/intermittent missing value,
4. consecutive missing values, and
5. entire feature missing.

**Figure 2:**
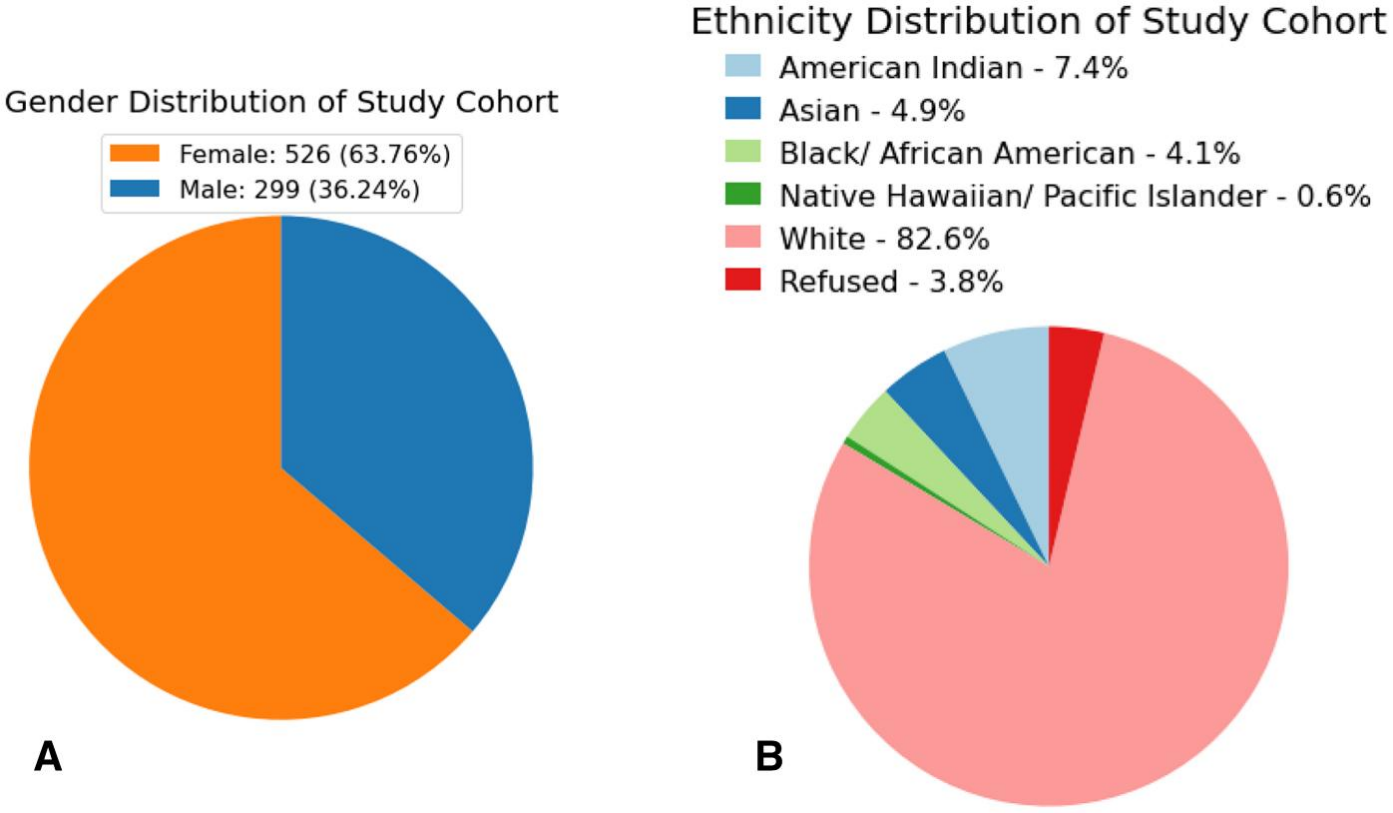
Distribution of (A) gender and (B) ethnicity in the study cohort.

We perform different strategies to resolve missing value issue for each of this scenarios, such as backfill, forward fill, linear interpolation, spline interpolation, and global mean fill. The final processed dataset has no missing values, information is sorted and stored according to their follow-up visit serials. Figure 2 shows the gender and ethnicity distribution in the study cohort.

#### Data standardization and padding for time-series compatibility

One key attribute of real-world clinical datasets is the variation in scale across different features. For instance, in healthy individuals, bilirubin levels typically range between 0.2 and 1.2 mg/dL, whereas alkaline phosphatase (ALP) levels range between 40 and 130 U/L. Such differences in measurement units and numerical magnitudes can bias model training and affect convergence. To address this, all features are standardized to a comparable scale, ensuring uniform contribution during training.

Additionally, since patients have varying numbers of follow-up visits, the dataset is padded to maintain consistent sequence lengths across all patients which is a necessary requirement for time-series analysis. Various padding strategies, such as interpolation, are used to achieve temporal alignment^16^; in our experiments, we padded with NaN (Not a Number) values and configured the model to treat these as placeholders rather than informative data points. This approach allows the model to disregard padded entries during training. Together, standardization and padding ensured that each feature contributed proportionally and that all time-series inputs were compatible for multivariate model learning.

### ML/DL models development for forecasting and NASH detection

#### Developing biomarker forecasting models and identifying minimum initial time steps required for optimal performance

**Figure 3:**
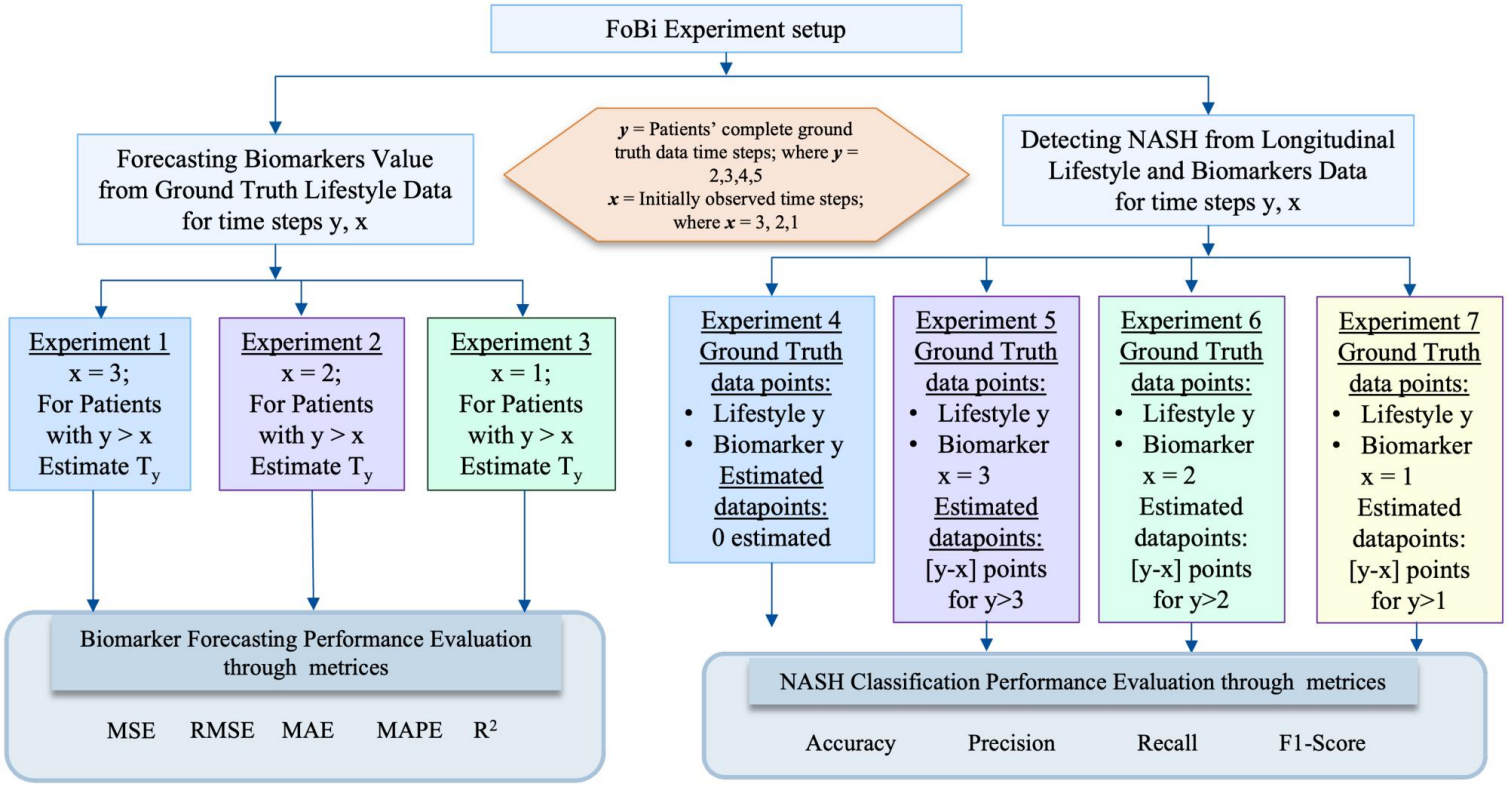
Experiment roadmap.

Figure 3 shows a brief overview of the experiments and data distribution we use for each task—biomarker forecasting and NASH detection. This phase focuses on forecasting future biomarker values from entire lifestyle data and a limited number of initial biomarker observations of the patients. We select the patient cohort based on the minimum number of their follow-up visits (minimum_followup_visit=2; and total_followup_visits_participants_have=y;where y≥2). Developing a multivariate time-series forecasting model requires fine-tuning and identifying appropriate hyperparameters for machine learning or deep learning models. Since our goal is to analyze the feasibility of NASH detection from *lifestyle data*, we prioritize interpretability and manually pick our features rather than relying solely on feature-selection techniques. The forecasting task of the *FoBi* method begins with experiment 1, where a minimum of 3 initially observed biomarker data points (x=3) are used to estimate the next (y−3) points, where *y* represents the total number of available data points per patient and y>x. We then evaluate the models’ forecasting performances while iteratively reducing the initially observed data points *x*, from 3 to 1 and predicting the next (y−x) data points. This design has 3 forecasting experiments (experiments 1-3), each exploring different input-output configurations.

For each experimental setup, several deep learning model, such as long short-term memory (LSTM), gated recurrent unit (GRU), and tree-based machine learning models like TimeSeriesForestRegressor (TSFR), diverse representation canonical interval forest (DrCIF) are trained and compared against a baseline model. We use naive estimation as baseline model for our work which is a standard practice in biomarker prediction tasks,^17^ providing a simple yet meaningful reference for evaluating model performance. We design the naïve baseline to predict the future biomarker values as constant at their most recent observed level, that is, $y_{t+1}=y_{t}$. This experimental framework allows us to assess not only forecasting performance but also to determine the minimum number of initial biomarker points required to achieve acceptable predictive performance.

#### Models developed for detecting NASH from the ground-truth scenario of biomarkers vs hybrid scenario of biomarkers

The second task evaluates whether the predicted biomarker trajectories (outputs from experiments 1-3) are sufficient for accurate disease classification for NASH. The *FoBi* methodology approaches this classification task in 2 direction: (1) ground-truth scenario: The classifier is trained and tested using all ground-truth biomarker values and corresponding lifestyle data to establish baseline detection performance (experiment 4). (2) Hybrid scenario: The classifier is trained using ground-truth biomarkers for initially observed data points, *FoBi*-estimated biomarker values for future points, and the complete lifestyle sequences to evaluate whether predicted biomarkers can maintain classification accuracy. We develop several models for this classification experiments, analyze, and compare their performance to identify the best-performing model. Performance of each classifier is evaluated using standard metrices, such as accuracy, precision, recall, F1-score, and regressor performance is evaluated using root mean square error (RMSE), normalized root mean square error (NRMSE), mean absolute error (MAE), $R^{2}$. These metrices allow comparison between NASH detection models built on real vs estimated biomarker trajectories.

## Results

### Naïve baseline performance

To establish a reference for evaluating the forecasting models, we first implement a naïve baseline that predicts future biomarker values as constant at their most recent observed level ($y_{t+1}=y_{t}$). Table 4 summarizes the baseline performance across 7 biomarkers, and Table 5 presents the corresponding SDs ($\sigma_{y}$) of the true biomarker values. The results in Table 4 show substantial variation in predictive accuracy, reflecting the heterogeneity in biomarker dynamics and scale. Among all biomarkers, direct bilirubin achieves the lowest error with an RMSE of 0.15 and an MAE of 0.09, indicating relative temporal stability and low variability (σ≈ 0.16). In contrast, biomarkers such as AST, GGT exhibit extremely high error magnitudes (RMSE>1700, MAPE>3800%), primarily due to their larger numerical ranges (AST range ≈15.7-46.9 U/L in healthy individuals,^18^ AST range ≈26–457 U/L for patients with liver cirrhosis^19^) and greater temporal fluctuations. Given the poor overall performance of the naïve estimator—especially for biomarkers with wide ranges, we decide not to use it as a baseline for subsequent analyses. Instead, among the 5 forecasting models developed (standard LSTM, GRU, Attention-LSTM, TSFR, and DrCIF), we choose the standard LSTM model as the comparative baseline. This decision ensures that all subsequent comparisons are made against a meaningful, data-driven benchmark rather than a static persistence model.

**Table 4:** Naïve baseline performance across biomarkers.

Biomarker	MSE	RMSE	MAE	NMSE	NRMSE	NMAE	MAPE (%)	$R^{2}$
**Total bilirubin**	0.3800	0.616	0.555	1.576	1.256	1.130	93.11	−0.576
**Direct bilirubin**	0.0225	0.150	0.095	0.907	0.952	0.601	50.01	0.093
**Albumin**	4.4419	2.108	2.069	18.076	4.252	4.174	52.04	−17.076
**AST**	2.93×10^6^	1710.74	1525.62	3249.08	57.00	50.83	3882.85	−3248.08
**ALT**	6.54×10^6^	2558.30	2218.19	5796.13	76.13	66.01	5304.79	−5795.13
**ALP**	1.12×10^7^	3349.21	3080.23	8172.13	90.40	83.14	3891.29	−8171.13
**GGT**	2.75×10^7^	5245.17	4177.28	14 009.67	118.36	94.26	8755.07	−14 008.67

Abbreviations: MSE, mean square error; RMSE, root mean square error; MAE, mean absolute error; NMSE, normalized mean square error; NRMSE, normalized root mean square error; NMAE, normalized mean absolute error; MAPE, Mean Absolute Percentage Error.

**Table 5:** Standard deviation of true biomarker values ($\sigma_{y}$) for experiment setup 1.

Biomarker	$\sigma_{y}$
**Total bilirubin**	0.491
**Direct bilirubin**	**0.157** ^a^
**Albumin**	0.496
**AST**	30.013
**ALT**	33.603
**ALP**	37.049
**GGT**	44.314

a direct bilirubin shows relative temporal stability and low variability (σ ≈ 0.16)

### Forecasting model performance and ground-truth comparison

**Figure 4:**
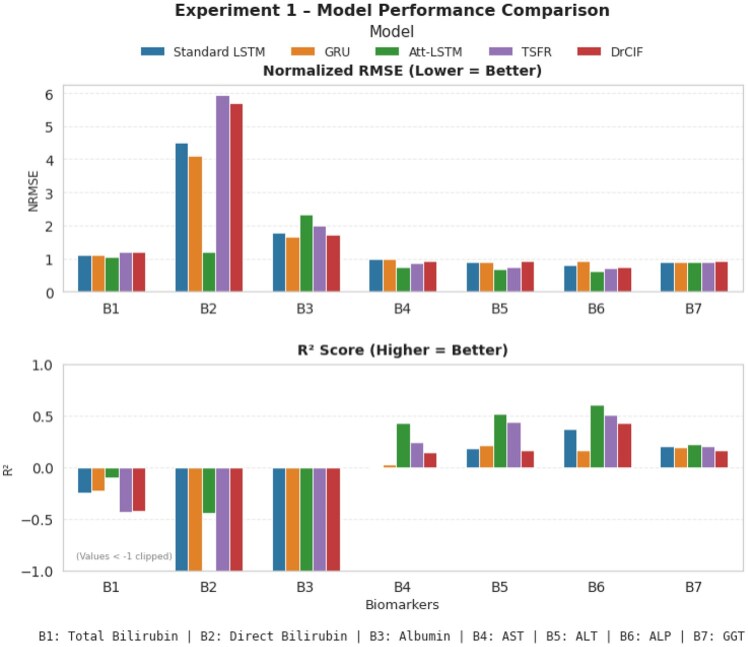
Comparison of forecasting model performance across liver biomarkers using normalized RMSE and *R*^2^ metrics in experiment 1 setup.

After completing each experiment of the forecasting task, we evaluate model performance using NRMSE and the coefficient of determination ($R^{2}$). For this task, models with performance [NRMSE<1]^20^ and $\left[R^{2}>0.2\right]$^21^ are considered stable and reliable for forecasting, representing strong alignment between predicted and actual biomarker trends. As shown in Figure 4, in experiment setup 1, the Attention-LSTM consistently achieves the best overall performance across biomarkers, exhibiting the lowest NRMSE values and highest $R^{2}$ scores for most biomarkers (B1, B2, B4-B6). The Standard LSTM performs competitively but slightly lags behind the Attention-LSTM, while the GRU achieves moderate performance. Among the tree-based approaches, the TSFR model performs close to the recurrent architectures, showing stable forecasts and comparable NRMSE. In contrast, the DrCIF model exhibits consistently higher error and greater instability. These results of experiment setup 1 demonstrate that both recurrent and ensemble-based sequence learners like TSFR can provide robust forecasts, with Attention-LSTM maintaining the best overall balance between accuracy and physiological interpretability.

**Figure 5:**
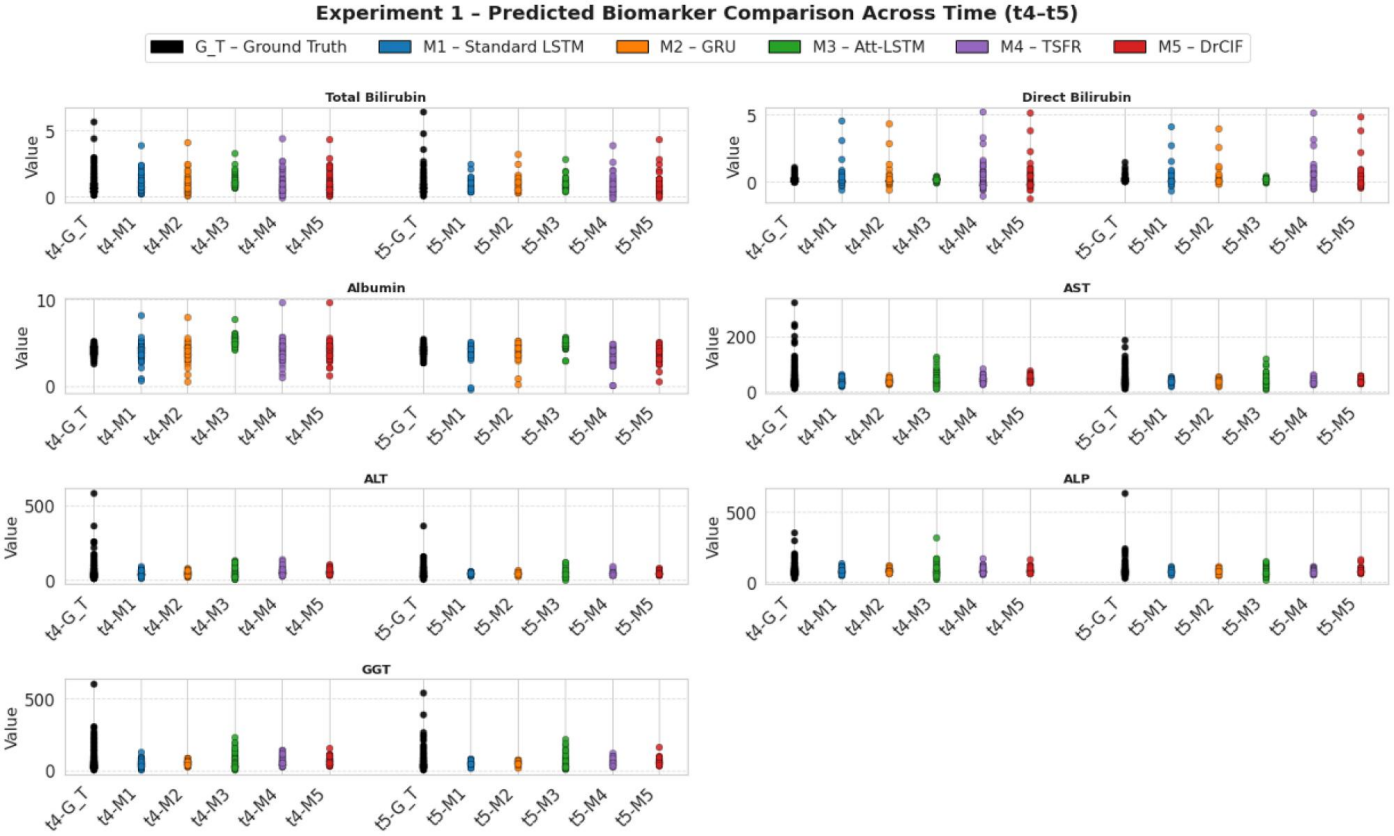
Experiment 1—Comparison of ground-truth and model-estimated biomarker values at timepoints t4 and t5 across 5 forecasting models.

Figure 5 presents predicted vs ground-truth biomarker distributions at timepoints $t_{4}$ and $t_{5}$ (experiment 1). For stable narrow range biomarkers (bilirubin, albumin), the predicted and observed values overlap closely, though statistical models occasionally produce slightly wider spreads than typically seen in clinical observations. For high-variance biomarkers (AST, GGT), all models show some underdispersion—that is, narrower prediction spread, but the Attention-LSTM maintains better alignment with observed ranges, effectively reproducing trend direction and amplitude despite noise.

Outliers are evident in the ground-truth data, particularly for AST, ALT, ALP, and GGT, corresponding to extreme enzyme elevations among patients with advanced disease. These outliers inflate aggregate error metrics and depress $R^{2}$ values for simpler models, yet both Attention-LSTM and Standard LSTM remain resilient, unaffected by the outliers, producing stable forecasts within physiologically plausible limits (which brings the coverage area down and NRME up, which looks like poor performance but apparently is not).

All the models we developed perform competitively. However, considering overall forecasting accuracy by $R^{2}$ consistency, error in prediction, and overlap with observed biomarker distributions, the *Attention-LSTM emerges as the best-performing model in experiment setup 1*, offering improved temporal sensitivity and stronger generalization to heterogeneous patient data when the model has seen initial biomarkers values for 3 consecutive visit.

**Figure 6:**
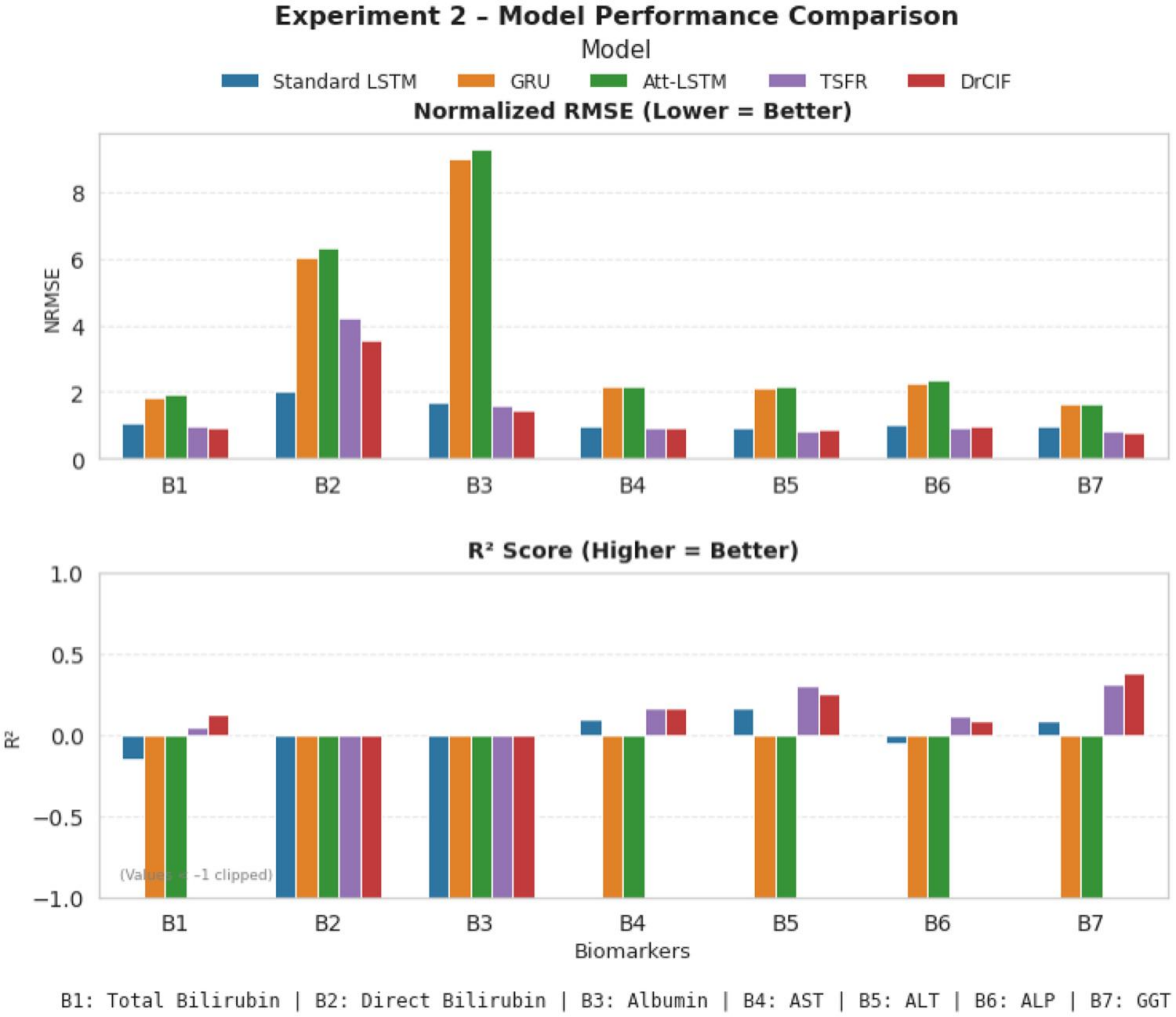
Comparison of forecasting model performance across liver biomarkers using normalized RMSE and $R^{2}$ metrics in experiment 2 setup.

We repeat the same performance analysis for experiment setups 2 and 3 to identify the best-performing model in those setups. In experiment 2, where models predict up to 3 future biomarker timepoints from 2 observed timepoints, the Standard LSTM and TSFR maintain their performance stability, while the Attention-LSTM, GRU, and DrCIF exhibit noticeable degradation in accuracy (Figure 6). This suggests that both Standard LSTM and TSFR can generalize effectively when temporal context is limited. In experiment 3, which relies on only one observed data point to forecast 4 future points, all models perform poorly—indicating that minimal historical context is insufficient for reliable multivariate forecasting. Overall, these findings suggest that *when large number of initial observations are available, Attention-LSTM remains the optimal choice due to its temporal weighting capability*; however, *as the number of observed points decreases, TSFR demonstrates greater robustness and stability under moderate temporal information conditions.* From these experiments, we determine that *a minimum of 2 observed biomarker timepoints is required to achieve acceptable forecasting accuracy for future biomarker estimation.* In addition to identifying optimal model architectures, these progressive experiments also serve as a robustness analysis under varying degrees of clinical supervision. By systematically reducing the number of initially observed biomarker timepoints (3, 2, and 1), we simulate real-world scenarios where laboratory data availability may be limited. The observed performance trends indicate that while forecasting accuracy degrades under extreme sparsity (single timepoint), models remain stable and generalizable when at least 2 prior biomarker observations are available. Importantly, robustness is further demonstrated by consistent behavior across both recurrent (LSTM-based) and ensemble (TSFR) models, as well as resilience to outliers present in this real-world longitudinal cohort. These conclusions directly guide the next phase-classification task. For experiment 5, we use estimated biomarker values only from Attention-LSTM (best performer in setup 1) and TSFR (stable across varying temporal contexts). For experiments 6 and 7, we exclusively use TSFR-generated estimates and discard the other models, which performed poorly in setups 2 and 3.

### NASH detection performance evaluation using observed and *FoBi*-estimated biomarkers

We next evaluate whether temporal patterns in biomarker trajectories—both clinically observed and *FoBi*-estimated can be used to detect NASH. 1D-CNN (One-Dimensional Convolutional Neural Network) architecture among our multivariate time-series classifiers trained on ground-truth biomarker sequences and complete lifestyle information achieves the strongest overall performance with an *accuracy of 0.76, precision of 0.78, recall of 0.71, and F1-score of 0.75*, outperforming the LSTM classifier (*accuracy of 0.66, F1-score of 0.67, AUC of 0.68*). These results demonstrate that temporal lifestyle patterns combined with full biomarker sequences contain sufficient discriminative structure for NASH detection, even without biopsy-specific alignment.

It is important to contextualize these results with prior work. The NASHmap study built on the same NIDDK dataset reports 70%-75% accuracy using a clean, biopsy-aligned cohort and a carefully curated 14-feature clinical panel collected on the biopsy date.^10^ Their dataset eliminates temporal variability by using only the biopsy-day measurement. In contrast, our dataset is substantially noisier: biomarker readings do not correspond to biopsy timepoints, the feature set is limited to 7, although we include full temporal lifestyle records to see if lifestyle information can be helpful in early liver disease detection. Despite this less controlled setting, our classifiers achieve performance in the same general range as NASHmap, demonstrating strong feasibility for a lifestyle-informed detection framework.

We then assess NASH detection using *FoBi*-estimated biomarker values (experiment 5). Using estimates generated by Attention-LSTM for patients with 4-5 samples (outputs from experiment 1), the 1D-CNN model achieves an *accuracy of 0.64, recall of 0.75, and F1-score of 0.71*, only moderately reduced from performance using only clinically observed biomarkers. From experiments 4 and 5, we can conclude that *even approximate biomarker trajectories derived from lifestyle information retain clinically meaningful signal for NASH classification*.

Finally, in experiment 8, we align our setup more closely with NASHmap^10^ by using only biomarker values collected on the biopsy day, paired with complete temporal lifestyle information. Under this configuration, our 1D-CNN model achieves its highest performance: *accuracy of 0.86, precision of 0.86, recall of 1.00, and F1-score of 0.92*, achieving competitive (and in our implementation, higher) performance than the originally reported baseline under this configuration. These results highlight 2 important insights: (1) lifestyle information provides additional discriminative value, exceeding performance obtained using biomarkers alone and (2) biopsy-aligned biomarkers carry the most definitive signal for NASH.

Taken together, the experiments demonstrate that (1) NASH can be detected from multivariate temporal lifestyle patterns combined with observed biomarkers; (2) *FoBi*-estimated biomarker trajectories are sufficiently accurate to support early stage detection; and (3) lifestyle information significantly enhances NASH detection performance, establishing a strong foundation for our future vision of a noninvasive NASH detection framework that operates primarily from lifestyle data and a minimal number of initial biomarker observations.

A key concern in 2-stage pipelines is the potential propagation of forecasting errors into downstream classification. Our hybrid evaluation (ground-truth vs *FoBi*-estimated biomarker trajectories) provides an explicit assessment of this effect. While performance modestly decreases when using estimated biomarkers, classification accuracy remains clinically meaningful and does not collapse under forecasting uncertainty. This stability suggests that *FoBi* preserves sufficient discriminative temporal structure despite approximation errors. Additionally, all NASH detection results are evaluated using 5-fold cross-validation, reducing the likelihood of overfitting and supporting model robustness. Collectively, these findings indicate that the framework generalizes across noisy, nonbiopsy-aligned real-world settings rather than relying on tightly curated clinical snapshots.

## Discussion

The forecasting experiments demonstrate that lifestyle information, combined with a limited number of initial biomarker observations, can reliably predict future biomarker trajectories. Models with temporal attention mechanisms, such as the Attention-LSTM, perform best when a large number of observed biomarker points are available. Meanwhile, tree-based models like *TSFR remain more robust under reduced temporal context*. These results collectively show that a *minimum of 2 observed biomarker values is sufficient for acceptable forecasting accuracy*, establishing a practical requirement for real-world use.

The NASH detection experiments highlight the potential of predictive value of lifestyle patterns when combined with longitudinal biomarker behavior. Our *classifier models trained on full ground-truth biomarker sequences and lifestyle data achieve performance comparable to the original biopsy-aligned work*. Importantly, *our NASH detection models trained on biomarker values that are FoBi-estimated, but designed and distributed similarly to the original study, achieve competitive performance within the range of previously reported results.* The *FoBi* framework is grounded in clinically validated endpoints, where NASH status is derived from biopsy-confirmed labels within the NIDDK NAFLD Adult Database, reflecting expert histopathological assessment of disease status. Additionally, the biomarkers modeled (ALT, AST, ALP, bilirubin, albumin, and GGT) correspond to standard liver function tests commonly interpreted in hepatology practice. Thus, while this work does not include prospective clinician-in-the-loop validation of model predictions, it is anchored in clinically established diagnostic measures. Future work will incorporate formal expert review and external multicenter validation to further strengthen translational readiness and clinical credibility. Given that our results suggest lifestyle information has significant impact on NASH detection, our future work will focus on developing a fully noninvasive NASH detection system driven primarily by lifestyle data. Ultimately, our goal is to build a lifestyle-centered monitoring framework capable of supporting early detection and long-term disease tracking in real-world settings. The progressive supervision experiments and hybrid biomarker evaluation collectively serve as indirect generalizability assessments. By evaluating performance under varying degrees of temporal sparsity and in both nonbiopsy-aligned and biopsy-aligned configurations, we simulate heterogeneous real-world deployment scenarios. Nevertheless, external validation on independent cohorts remains an important future direction to further confirm cross-population generalization.

## Conclusion

We presented *FoBi*, a 2-phase framework that leverages longitudinal lifestyle information and a minimal number of initial biomarker measurements to (1) forecast future liver biomarker trajectories and (2) detect NASH using either observed or *FoBi*-estimated biomarker sequences together with lifestyle data. Using the NIDDK NAFLD Adult Database, we showed that reliable multibiomarker forecasting is achievable when at least 2 prior biomarker timepoints are available; model choice should be conditioned on the amount of temporal context, with Attention-LSTM performing best when richer biomarker history is present and TSFR offering greater robustness when observations are limited. We further demonstrated that NASH classification remains feasible in both real-world settings with nonbiopsy-aligned longitudinal measurements and in a biopsy-aligned configuration, and that *FoBi*-estimated biomarker trajectories preserve clinically meaningful signal for downstream detection. Collectively, these findings support the feasibility of minimally invasive, lifestyle-informed monitoring pipelines that can reduce dependence on frequent laboratory testing and enable longitudinal tracking of NAFLD progression.

Future work will focus on improving generalization across clinical settings, strengthening interpretability, and advancing toward a fully noninvasive lifestyle-centered NASH detection and monitoring system.

## Data Availability

No new data were collected in support of this research. The NAFLD Adult Database study was conducted by the NASH Clinical Research Network (CRN) Investigators and supported by the National Institute of Diabetes and Digestive and Kidney Diseases (NIDDK). The data from the NAFLD Adult Database used here were supplied by the NIDDK Central Repository. This manuscript was not prepared in collaboration with NASH CRN Investigators and does not necessarily reflect the opinions or views of the NASH CRN Investigators, the NIDDK Central Repository, or the NIDDK.
